# 148. Single-amplicon, Multiplex Real-time RT-PCR with Tiled Probes to Detect SARS-CoV-2 *spike* Mutations Associated with Variants of Concern

**DOI:** 10.1093/ofid/ofab466.148

**Published:** 2021-12-04

**Authors:** Maxwell Su, Katherine S Immergluck, Samuel Stampfer, Anuradha Rao, Leda Bassitt, Vi Nguyen, Victoria D Stittleburg, Jessica M Ingersoll, Colleen S Kraft, Greg S Martin, Anne Piantadosi, Wilbur A Lam, Jesse Waggoner, Ahmed Babiker

**Affiliations:** 1 Emory University School of Medicine, Atlanta, GA; 2 Emory University School of Medicine, Atlanta, Georgia; 3 Emory University, Atlanta, Georgia

## Abstract

**Background:**

Detection and surveillance of severe acute respiratory syndrome coronavirus 2 (SARS-CoV-2) variants is of great public health importance. Broadly accessible and inexpensive assays are needed to enhance variant surveillance and detection globally. We developed and validated a single-reaction multiplex real-time RT-PCR (the Spike SNP assay) to detect specific mutations associated with variants of concern (VOC).

**Methods:**

A single primer pair was designed to amplify a 348 bp region of *spike*. Probes were initially designed with locked nucleic acids (LNAs) to increase probe melting temperature, shorten probe length, and specifically detect 417K, E484K, and N501Y (**Figure**). The assay was optimized and evaluated using characterized variant sample pools. Clinical evaluation was performed on a convenience set of residual nasopharyngeal swabs, and variant calls were confirmed by SARS-CoV-2 genomic sequencing in a subset of samples. Following the initial evaluation, unmodified probes (without LNAs) were designed to detect L452R, L452Q, and E484Q.

Figure. Spike SNP distinguishes mutations occurring in different lineages (A-C).

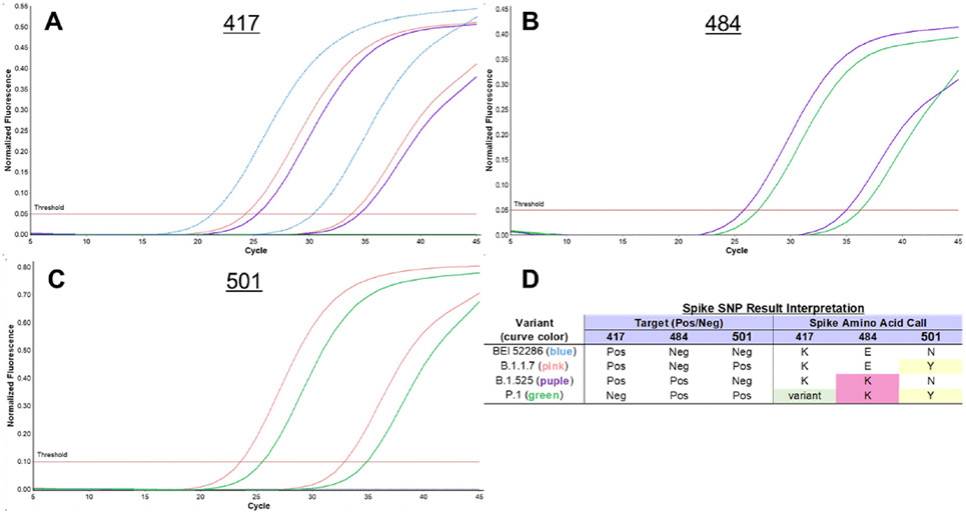

Representative results of variant detection a single Spike SNP run are shown for mutations in the codons for 4177K (A) and mutations that encode 484K (B) and 501Y (C). Curves show dilutions of the following variants: blue, BEI 52286 (wild type); pink B.1.1.7; purple, B1.525; and green, P.1. Variant pools were used for B.1.17, B.1.525, and P.1 strains. Curves are displayed for a given dilution in each channel and result interpretation is shown (D).

**Results:**

The lower limit of 95% detection was 2.46 to 2.48 log_10_ GE/mL for the three targets (~1-2 GE/reaction). Among 253 nasopharyngeal swabs with detectable SARS-CoV-2 RNA, the Spike SNP assay was positive in 238 (94.1%), including all samples with Ct values < 30 (220/220) for the N2 target and 18/33 samples with N2 Ct values ≥ 30. Results were confirmed by SARS-CoV-2 genomic sequencing in 50/50 samples (100%). Subsequent addition of the 452R probe did not affect performance for the original targets, and probes for 452Q and 484Q performed similarly to LNA-modified probes.

**Conclusion:**

The Spike SNP assay provides fast, inexpensive and sensitive detection of specific mutations associated with SARS-CoV-2 VOCs, and the assay can be quickly modified to detect new mutations in the receptor binding domain. Similar analytical performance of LNA-modified and unmodified probes presents options for future assay customization that balance the shorter probe length (LNAs) and increased accessibility (unmodified). The Spike SNP assay, if implemented across laboratories offering SARS-CoV-2 testing, could greatly increase capacity for variant detection and surveillance globally.

**Disclosures:**

**Colleen S. Kraft, MD, MSc**, Rebiotix (Individual(s) Involved: Self): Advisor or Review Panel member

